# New species of *Triplocania* Roesler (Psocodea, ‘Psocoptera’, Ptiloneuridae), from Brazil and Ecuador

**DOI:** 10.3897/zookeys.505.9870

**Published:** 2015-05-25

**Authors:** Alberto Moreira Da Silva Neto, José Albertino Rafael, Alfonso N. García Aldrete

**Affiliations:** 1 Instituto Nacional de Pesquisas da Amazônia – INPA, CPEN – Programa de Pós-Graduação em Entomologia, Campus II, Caixa postal 478, CEP 69011-97, Manaus, Amazonas, Brasil; 2 Departamento de Zoología, Instituto de Biología, Universidad Nacional Autónoma de México, Apartado Postal 70-153, 04510 México, D. F., MÉXICO

**Keywords:** Epipsocetae, taxonomy, neotropics

## Abstract

Four species of *Triplocania*, three with M3 simple, based on male specimens and one with forewing M3 forked, based on male and female specimens, are here described and illustrated, namely: *Triplocania bravoi* **sp. n.** (Napo: Ecuador), *Triplocania erwini* **sp. n.** (Napo: Ecuador), *Triplocania trifida* **sp. n.** (Mato Grosso and Rondônia: Brazil) and *Triplocania lamasoides* **sp. n.** (Rondônia: Brazil). They differ from all the other species in the genus, in which the males are known, by the hypandrium and phallosome structures. The female is first described for the M3 forked group. The identification key for males of the M3 forked group is updated.

## Introduction

*Triplocania* Roesler (1940) is one of 12 genera in the psocopteran family Ptiloneuridae; it is the most species rich genus of this family. It presently includes 32 described species that, according to forewing venation, can be separated in two groups: a large one with 25 species, characterized by having forewing venation caeciliusid, that is, with Rs of two branches, and with M of three branches, this group is here referred as MPB group (M with only primary branches); and a smaller group with 7 species, characterized by having M with three primary and secondary branches, this group is here referred as MSB group (M with secondary branches); this group is divided in two subgroups: the first (MSB1) is represented by *Triplocania palaciosi* García Aldrete & Casasola González (2012), it is characterized by having more than one M vein with secondary branches, the branches originating closer to the wing margin than to the main M. The second subgroup (MSB2) is represented by 6 species; it is characterized by having only one secondary branch, in M_3_, resulting in M_3a_ and M_3b,_ and with branches originating closer to the main M than to the wing margin. The purpose of this work is to describe and illustrate three new species of *Triplocania* belonging in group MPB, based on males, and to describe a new species belonging in subgroup MSB2 mentioned above, based on males and females.

## Material and methods

Ten specimens were available for study; they were dissected in 80% ethanol; their parts (head, right legs and wings, and genitals) were mounted in Canada balsam. Before dissecting, whole specimens were placed in 80% ethanol under a dissecting microscope, illuminated with cold, white light, and observed at 50× to record color. Standard measurements (in μm), were taken with a filar micrometer. Abbreviations of parts measured are as follows: FW and HW: right fore- and hind- wing length, F, T, t1, t2 and t3: lengths of femur, tibia and tarsomeres 1, 2 and 3 of right hind leg, f1…fn: lengths of flagellomeres 1…n of right antenna, Mx4: length of fourth segment of right maxillary palpus, IO: minimum distance between compound eyes in dorsal view of head, D and d: antero-posterior and transverse diameter, respectively, of right compound eye in dorsal view of head, PO: d/D. The types of the Brazilian species will be deposited in the Invertebrate Collection of the Instituto Nacional de Pesquisas da Amazônia (INPA), Manaus, Amazonas, Brazil. The types of the Ecuadorian species will be deposited in the Sección de Entomología, Instituto de Ciencias Biológicas, Escuela Politécnica Nacional, in Quito, Ecuador (EPN).

## Taxonomy

### Triplocania bravoi sp. n.

Taxon classificationAnimaliaPsocodeaPtiloneuridae

http://zoobank.org/C852F46E-918E-47ED-8C24-F618303BA703

Figures 1–7

#### Type-locality.

Ecuador, Napo: Reserva Étnica Waorani, 1 Km S. Onkone Gare Camp, 220m, 0°30'10"S, 76°26'0"W, fogging terre firma forest, 12.II.1995, T. L. Erwin et al. leg.

#### Type-material.

Holotype male, mounted on one slide; thorax in a separate microvial. Original label: Ecuador. Napo. Reserva Étnica Waorani, 1 Km S. Onkone Gare Camp. 220m. 12.II.1995. 0°30'10"S: 76°26'0"W. Fogging terre firma forest. T. L. Erwin et al. (EPN, slide 160, vial 160).

#### Etymology.

This species is dedicated to the Ecuadorian entomologist Freddy Rubén Bravo Quijano, of the Universidade Estadual de Feira de Santana, Bahia, Brazil, in recognition to his important contributions in the taxonomy of Neotropical Psychodidae (Diptera), also for the support to AMSN, to pursue a career studying Psocodea, ‘Psocoptera’.

#### Diagnosis.

Differing from the known species of *Triplocania*, in having the hypandrium with side sclerites fused proximally to the central piece, and having two forked posterior projections, horn shaped; also by having a U–shaped phallosome, a phallobase with lateral extensions covering partly the anterior pairs of endophallic sclerites, and in having ornamented areas on the endophallus.

#### Male.

**Figures 1–7.**
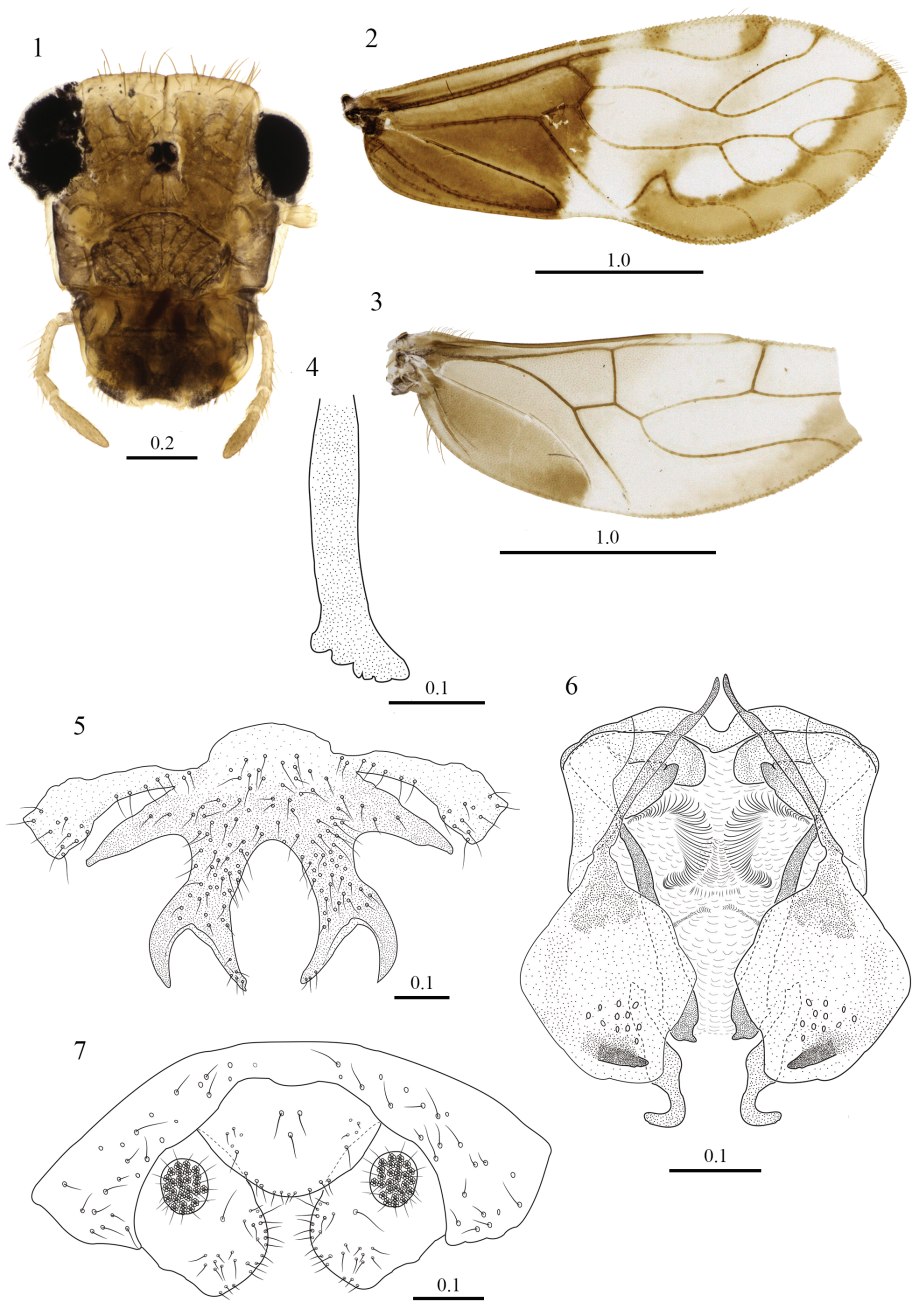
*Triplocania bravoi* sp. n. (Holotype male). **1** Front view of head **2** Forewing **3** Hindwing **4** Lacinial tip **5** Hypandrium **6** Phallosome in dorsal view **7** Clunium, paraprocts and epiproct. Scales in mm.

**Color.** Compound eyes black, ocelli hyaline, with ochre centripetal crescents; head pattern (Fig. 1). Scape and pedicel pale yellow, f_1_–f_3_ pale yellow, with apex white. Mx4 pale brown. Femora pale yellow; tibiae yellow, distally pale brown; tarsomere 1 pale yellow, tarsomeres 2–3 pale brown. Forewing with a brown marginal band from R_4+5_ to almost CuA_2_; a brown, almost rectangular band, well pigmented proximally and distally, weakly pigmented in the middle, from the apex of the areola postica to posterior end of the pterostigma; pterostigma with proximal and distal brown bands. Proximal third of forewing dark brown, limited posteriorly by level of crossvein Rs–M, as illustrated (Fig. 2). Veins brown. Hindwing with area below CuP dark brown, and a marginal pale brown band from R_4+5_ to almost CuA (Fig. 3).

#### Morphology.

As in diagnosis, plus the following: compound eyes without interommatidial setae. Outer cusp of lacinial tip broad, with five denticles (Fig. 4). Forewing pterostigma long, widest in the middle. Areola postica very wide basally, slanted posteriorly, tall proximally with apex round and narrow, distally sinuous and low; R_2+3_ and R_4+5_ sinuous, M stem slightly concave proximally, M_1,_ M_2_ and M_3_ sinuous. Hindwing Rs almost straight, R_2+3_ straight, R_4+5_ almost straight, M sinuous. Hypandrium of three sclerites, central piece anteriorly convex, with side projections almost parallel to side sclerites, forked posterior projections densely setose, other setae as illustrated (Fig. 5). Phallosome U-shaped, side struts independent, V shaped, fused posteriorly to external parameres, these stout, bearing a field of pores distally, each with an small projection heavily sclerotized distally; three pairs of endophallic sclerites, a posterior pair, curved outwards distally, close to the ends of the external parameres, a mesal pair, originating from behind the side struts, crossing behind the external parameres, distally dilated, with a small, acuminate projection apically; anterior pair stout, curved inwards, apically pointed and proximally rounded; phallobase with anterior border concave; lateral extensions covering partly the anterior pair of endophallic sclerites; endophallus membranous, with areas thickened and ornamented as illustrated (Fig. 6). Paraprocts broad, a field of setae along inner margin, other setae on apex; sensory fields with 27–28 trichobothria on basal rosettes (Fig. 7). Epiproct wide based, posteriorly rounded, with three large mesal setae, next to anterior margin, other setae as illustrated (Fig. 7).

#### Measurements

(in microns). FW: 3289, HW: 2108, F: 774, T: 1377, t1: 285, t2: 59, t3: 117, f1: 466, f2: 397, f3: 270, Mx4: 201, IO: 440, D: 332, d: 186, PO: 0.56.

### Triplocania erwini sp. n.

Taxon classificationAnimaliaPsocodeaPtiloneuridae

http://zoobank.org/4269F9B2-0360-4006-BD6D-9CBFA4872294

Figures 8–14

#### Type-locality.

Ecuador, Napo: Reserva Étnica Waorani, 1 Km S. Onkone Gare Camp, 220m, 0°30'10"S: 76°26'0"W, fogging terre firma forest, 12.II.1995, T. L. Erwin et al. leg.

#### Type-material.

Holotype male, mounted on slides; thorax in a separate microvial. Original label: Ecuador. Napo. Reserva Étnica Waorani, 1 Km S. Onkone Gare Camp. 220m. 12.II.1995. 0°30'10"S: 76°26'0"W. Fogging terre firma forest. T. L. Erwin et al. Paratype: 1 male, same data as the holotype (EPN, slides 163–164, vials 163–164).

#### Etymology.

This species is dedicated to Dr. Terry L. Erwin, of the Smithsonian Institution, Washington, D. C., USA., in recognition to his seminal studies in biodiversity, in estimating the number of arthropods on this planet, in systematics and biology of the Carabidae, and for making available for study to ANGA, the psocid specimens collected by his team in Napo, Ecuador, by canopy fogging.

#### Diagnosis.

Differing from the known species of *Triplocania*, in having the hypandrium with side sclerites and central piece similar in size; central piece with two short, lateral posterior projections, and two short, blunt ended, median posterior projections, leaving between them a small concavity, in having the external parameres with a distinct lobe apically on the inner side and, in having two pairs of endophallic sclerites.

#### Male.

**Figures 8–14.**
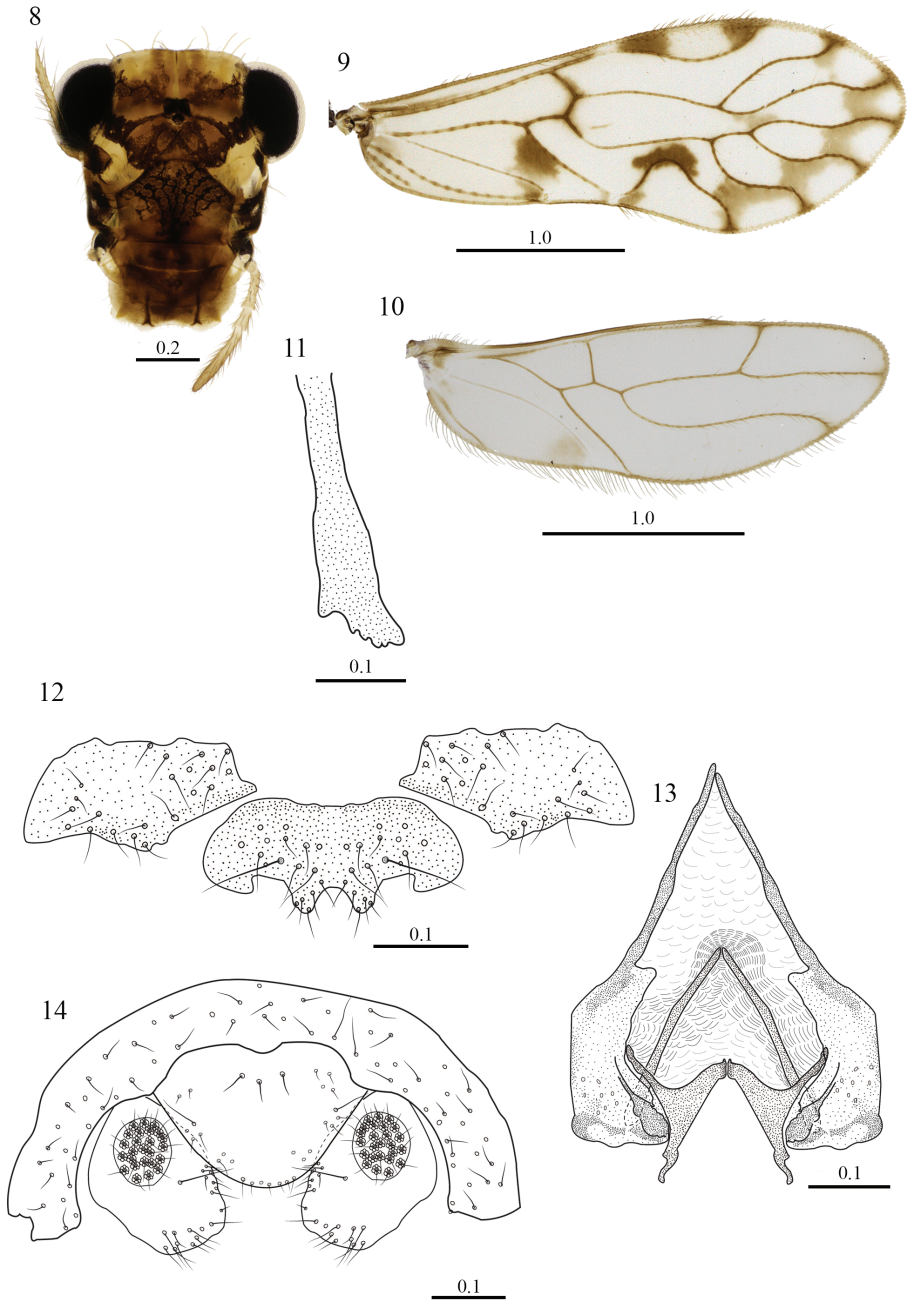
*Triplocania erwini* sp. n. (Holotype male). **8** Front view of head **9** Forewing **10** Hindwing **11** Lacinial tip. **12** Hypandrium **13** Phallosome in dorsal view **14** Clunium, paraprocts and epiproct. Scales in mm.

**Color.** Body pale brown, with ochre spots as indicated below. Compound eyes black, ocelli hyaline, with ochre centripetal crescents; head pattern (Fig. 8). Scape brown and pedicel yellow, f_1_–f_3_ yellow, with apices white. Mx4 brown. Tergal lobes of meso and methathorax brown, pleura with ochre spots above the level of the coxae; dark brown bands on proximal and distal ends of coxae, femora yellow with three brown equidistant bands, a middle one, and one on each end of the femur; tibiae pale brown, tarsomeres 1–3 yellow. Forewing with an irregular, submarginal brown band from R_2+3_ to areola postica, this with a small brown spot proximally, and a dark brown spot between its apex and M, a dark brown spot below the proximal end of CuA, and a brown spot at confluence of CuP–1A; a pale brown spot between proximal ends of R_4+5_–M_1_; pterostigma with brown bands anteriorly and posteriorly; veins brown, with brown spots at wing margin (Fig. 9). Hindwing almost hyaline, veins brown, with a pale brown spot at confluence of CuP and wing margin (Fig. 10).

#### Morphology.

As in diagnosis plus the following: compound eyes without interommatidial setae. Outer cusp of lacinial tip broad, with five denticles (Fig. 11). Forewing pterostigma basally narrow, wider in the middle; areola postica wide basally, slightly slanted posteriorly; R_2+3_ and R_4+5_ sinuous, M stem slightly concave proximally, M_1,_ M_2_ and M_3_ sinuous. Hindwing Rs almost straight, R_2+3_ and R_4+5_ straight, M sinuous. Hypandrium with two large setae posteriorly between each lateral-median posterior projections (Fig. 12). Phallosome with side struts independent, V shaped, fused posteriorly to external parameres, these stout, with pores posteriorly; the distal lobe of each heavily sclerotized; anterior pair of endophallic sclerites elongate, almost touching anteriorly, inserted on a membranous, V shaped, thickened area, lying distally behind the external parameres, ending in a rounded apex; posterior pair of endophallic sclerites triangular, each anteriorly concave, close to the inner border of the external parameres, with apices sinuous.

#### Measurements

(in microns). FW: 3302, HW: 2270, F: 645, T: 882, t1: 330, t2: 63, t3: 115, f1: 519, f2: 384, f3: 330, Mx4: 239, IO: 398, D: 326, d: 194, PO: 0.59.

### Triplocania lamasoides sp. n.

Taxon classificationAnimaliaPsocodeaPtiloneuridae

http://zoobank.org/604C9997-0C41-4894-9C41-0B16CCE7CFFF

Figures 15–21 , 22–26

#### Type-locality.

Brazil, Rondônia: Ariquemes, Rio ji Paraná, 90°44'S: 61°52'W, Malaise trap. 28.I.1986, J. A. Rafael leg.

#### Type-material.

Holotype male, mounted on slides, with thorax in a separate microvial. Original label: Brasil. Rondonte [Rondônia]. Ariquemes, Rio ji Paraná. 28.I.1986. 90°44'S: 61°52'W. Malaise trap. J. A. Rafael. Paratypes: 1 female and 3 males, same data as the holotype (INPA, slides 57–61, vials 57–61).

#### Etymology.

The specific name refers to the proximity of this species to *Triplocania lamasi* Silva–Neto, Rafael & García Aldrete.

#### Diagnosis.

Differing from *Triplocania lamasi* in having the posterior sclerite of the hypandrium thicker in the middle, with the posterior projection more than twice as long; sickle-shaped lateral projections distal to the anterior sclerite barely reaching the inner margins of the lateral sclerites.

#### Male.

**Figures 15–21.**
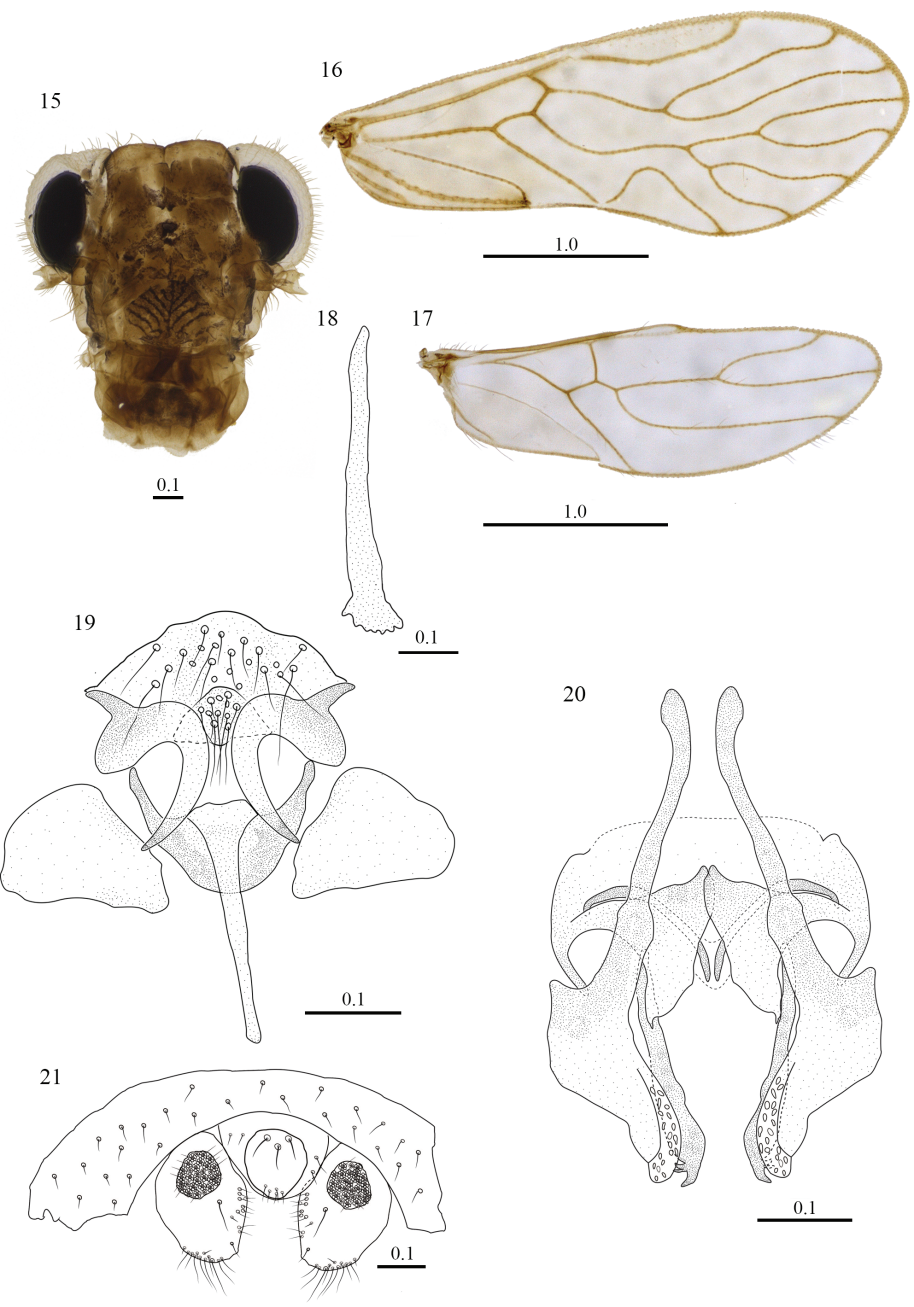
*Triplocania lamasoides* sp. n. (Holotype male). **15** Front view of head **16** Forewing **17** Hindwing **18** Lacinial tip **19** Clunium, paraprocts and epiproct **20** Hypandrium **21** Phallosome in dorsal view. Scales in mm.

**Color.** Body yellowish brown, with dark brown spots as indicated below. Compound eyes black, ocelli hyaline, with ochre centripetal crescents; head pattern (Fig. 15). Scape and pedicel pale brown; flagellomeres pale yellow. Mx4 pale yellow. Tergal lobes of meso- and metathorax reddish brown; episternum of mesothorax ochre. Coxae, trochanters and femora creamy white, tibiae and tarsomeres pale yellow. Forewings hyaline, as illustrated (Fig. 16); veins brown. Hindwing (Fig. 17), hyaline throughout, veins brown.

#### Morphology.

As in diagnosis, plus the following: compound eyes with interommatidial setae. Outer cusp of lacinial tip broad, with six denticles (Fig. 18). Forewing pterostigma long, widest in the middle. Areola postica wide basally, slightly slanted posteriorly, apex round, narrow. R_2+3_ and R_4+5_ sinuous, M stem concave, M_1_ almost straight, M_2_ sinuous, M_3_ branched, the branching point closer to M than to the wing margin. Hindwing Rs almost straight. Hypandrium (Fig. 19) of four sclerites, anterior piece broad, setose, bearing distally two sickle-shaped lateral projections, heavily sclerotized at both ends, and having also a well defined, setose sclerotized area in the middle; posterior sclerite concave anteriorly, with a long, slender posterior projection in the middle, flanked by two large, broadly triangular lateral sclerites. Phallosome (Fig. 20) with side struts independent, V shaped, fused posteriorly to external parameres, these stout, each with an elongate projection on inner margin, with field of pores; three pairs of endophallic sclerites; anterior pair long, slender and curved, mesal pair wide proximally, narrowing distally, pointed, and posterior pair parallel to the inner margin of the external parameres, with three acuminate projections distally. Paraprocts broad, wide proximally, narrowing to round apex; with a field of short setae along inner margin, other setae as illustrated; sensory fields with 30–31 trichobothria on basal rosettes (Fig. 21). Epiproct mesally with an almost elliptic protuberance, with a field of setae posteriorly, and three large mesal setae next to anterior margin (Fig. 21).

#### Measurements

(in microns). FW: 3710, HW: 2465, F: 910, T: 1493, t1: 622, t2: 77, t3: 132, f1: 556, f2: 455, f3: 390, Mx4: 170, IO: 470, D: 395, d: 210, PO: 0.53.

#### Female.

**Color.** Essentially as in the male.

#### Morphology.

**Figures 22–26.**
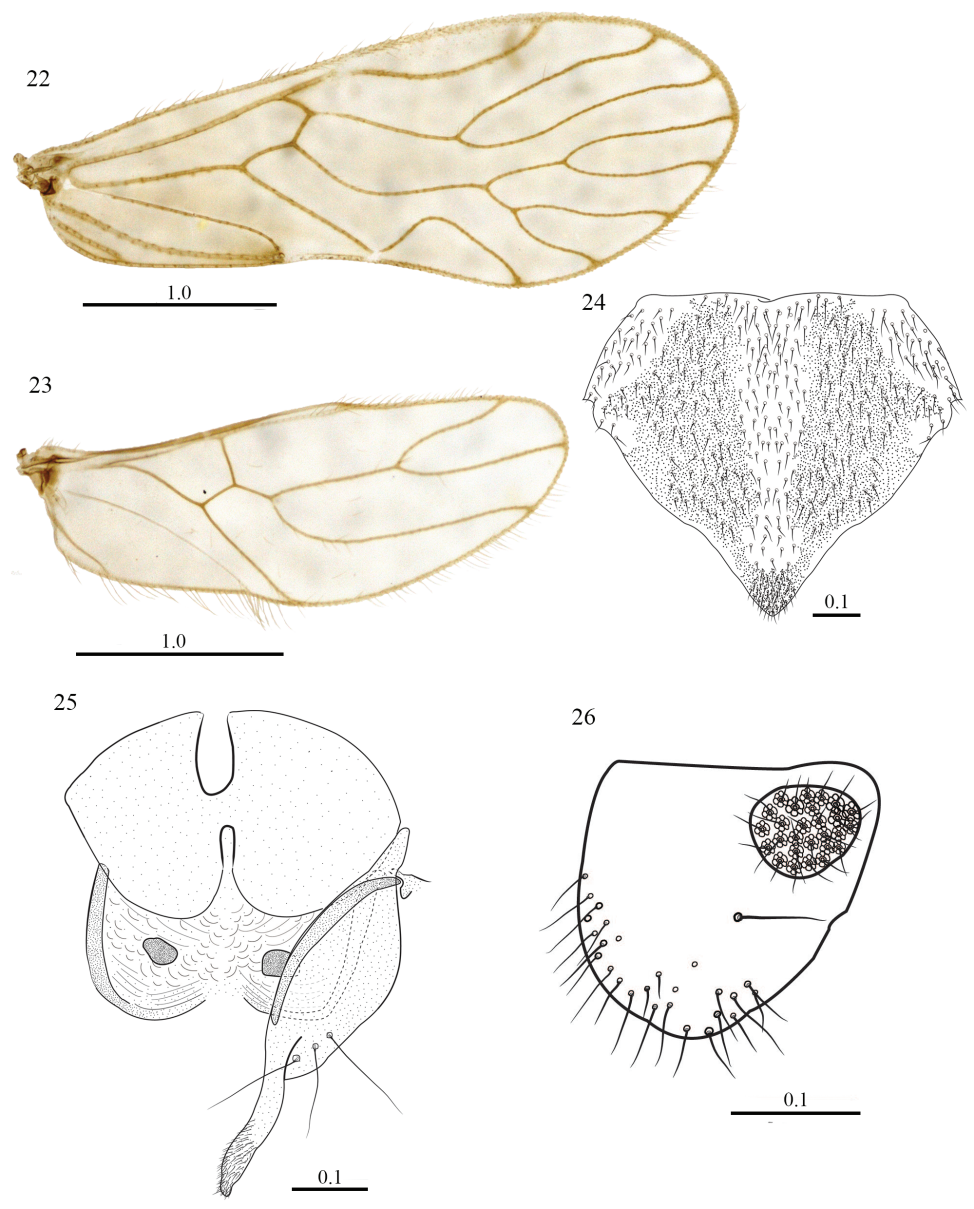
*Triplocania lamasoides* sp. n. (Paratype female). **22** Forewing **23** Hindwing **24** Subgenital plate **25** Gonapophyses and Ninth sternum **26** Left paraproct Scales in mm.

Fore- and hind- wings (Figs 22, 23) same as in the male. Subgenital plate broad, V shaped, pigmented area wide, setae as illustrated (Fig. 24); Gonapophyses: V_1_ long, slender, heavily sclerotized; V_2+3_ stout, heeled, narrow anteriorly and wider in the middle, with three large setae on outer lobe as illustrated, distal process stout, sinuous, distally blunt, with a field of microsetae (Fig. 25). Ninth sternum broad, with two distinct areas, the anterior one unpigmented, with a concavity anteriorly and posteriorly in the middle; posterior area pigmented, thicker than the anterior one, with a strongly sclerotized band latero-posteriorly, and a small, strongly pigmented area mesally on each side. Paraprocts broad, almost triangular, wide proximally, narrowing to round apex, setose posteriorly as illustrated, sensory fields with 26–27 trichobothria on basal rosettes (Fig. 26). Epiproct missing.

#### Measurements

(in microns). FW: 3723, HW: 2560, F: 890, T: 1385, t1: 607, t2: 58, t3: 121.

### Triplocania trifida sp. n.

Taxon classificationAnimaliaPsocodeaPtiloneuridae

http://zoobank.org/B47478F7-C56A-4B90-9179-EBB42A829071

Figures 27–33

#### Type-locality.

Brazil, Mato Grosso: Chapada dos Guimarães, 23-30.XI.1983. A. Yamamoto leg.

#### Type-material.

Holotype male, mounted on slides; thorax in a separate microvial. Original label: Brasil. Mato Grosso. Chapada dos Guimarães. 23-30.XI.1983. A. Yamamoto. Paratypes: 1 male, Original label: Brasil. Rondonte [Rondônia]. Ariquemes. Rio ji Paraná. 28.I.1986. 09°44'S: 61°52´W. Malaise trap. J. A. Rafael. (INPA, slides 112–113, vials 112–113).

#### Etymology.

The specific name refers to the characteristic of the hypandrium, having three posterior projections.

#### Diagnosis.

Differing from the known species of *Triplocania*, in having the central piece of the hypandrium with three posterior projections, a middle one, pointed, setose, flanked by lateral, strongly sclerotized, glabrous acuminate projections. Phallosome with a transverse, strongly sclerotized mesal bridge, biconcave anteriorly, convex posteriorly, widest in the middle, narrowing to the sides; four pairs of endophallic sclerites; external parameres distally with an elliptic papillose field.

#### Male.

**Figures 27–33.**
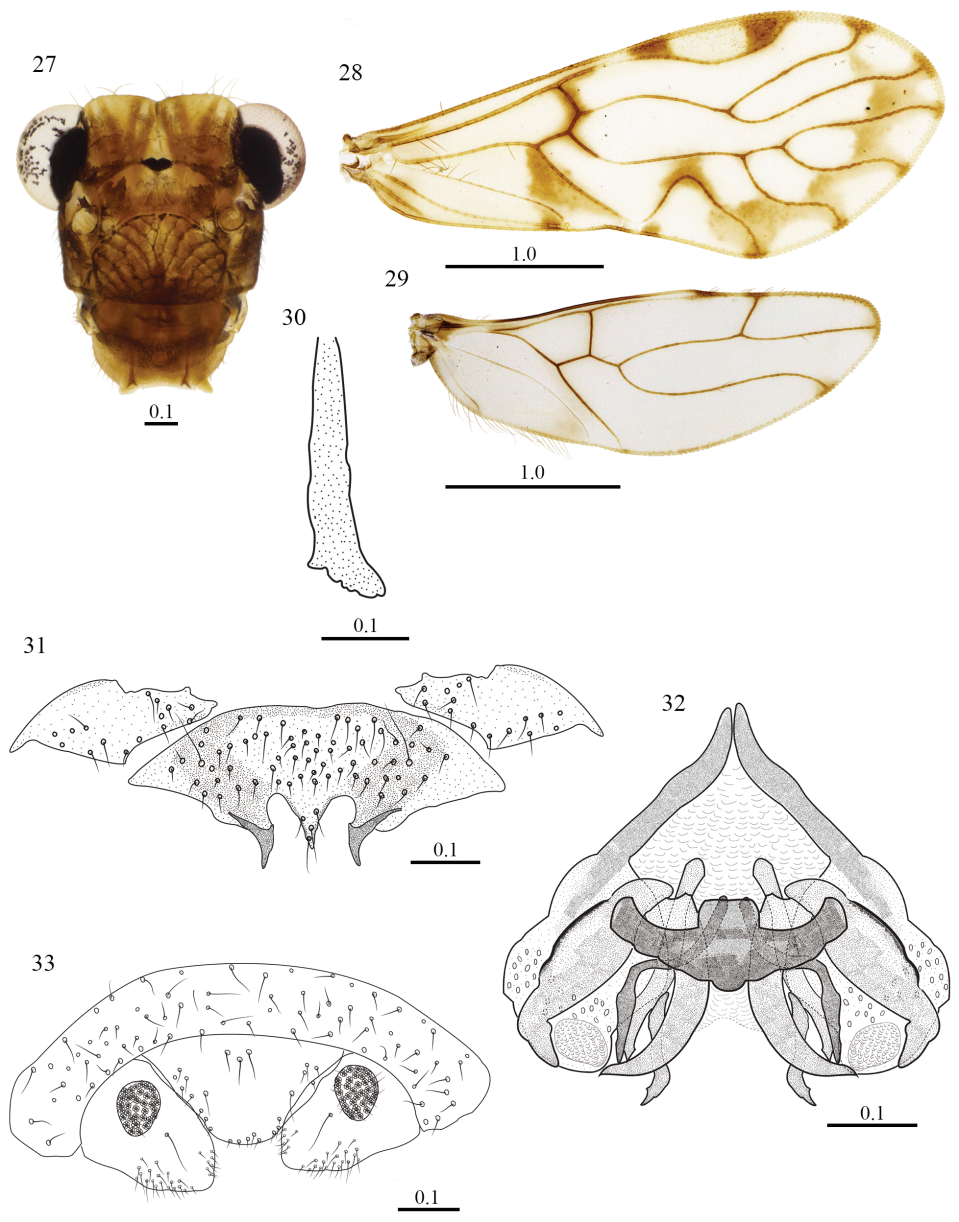
*Triplocania trifida* sp. n. (Holotype male). **27** Front view of head **28** Forewing **29** Hindwing **30** Lacinial tip **31** Hypandrium **32** Phallosome in dorsal view **33** Clunium, paraprocts and epiproct. Scales in mm.

**Color.** Body yellow, with ochre spots as indicated below. Compound eyes black, ocelli hyaline, with ochre centripetal crescents. Head pattern (Fig. 27). Scape brown, pedicel pale brown, f_1_ anteriorly pale brown, posteriorly yellow, apex white, f_2_ yellow. Mx4 brown. Tergal lobes of meso and methathorax pale brown, pleura yellow; femora pale yellow, tibiae pale brown, tarsomeres 1–3 yellow. Forewing with an irregular, submarginal pale brown band from R_2+3_ to posterior end of areola postica, this with a small brown spot proximally, and a dark brown spot between its apex and M; a triangular brown area next to CuA, and a brown spot at confluence of CuP–1A; pterostigma with brown bands anteriorly and posteriorly; veins brown, with brown spots at wing margin (Fig. 28). Hindwing almost hyaline, with small brown spots distally on veins M, R_2+3_ and R_4+5;_ with a pale brown spot at confluence of CuP and wing margin; veins brown (Fig. 29).

#### Morphology.

As in diagnosis, plus the following: compound eyes without interommatidial setae. Outer cusp of lacinial tip broad, with five denticles (Fig. 30). Forewing pterostigma wider in the middle, narrow anteriorly; Rs convex, R_2+3_ almost straight proximally and concave distally, R_4+5_ sinuous, M stem slightly concave proximally, then almost straight, M_2_–M_3_ sinuos, areola postica wide basally, slightly slanted posteriorly; hindwing Rs almost straight, R_2+3_ and R_4+5_ straight, M sinuous. Hypandrium of three sclerites; side sclerites, large, irregular, with setae as illustrated (Fig. 31). Phallosome with side struts independent, V shaped, fused posteriorly to external parameres, these stout, with pores posteriorly. Anterior pair of endophallic sclerites elongate, sinuous, distally acuminate; a central pair, narrow, elongate, sinuous, heavily sclerotized, with anterior end blunt, posteriorly dilated, lance-shaped; a lateral pair biramous, with inner arms long, curved out, wide based, extended posteriorly and distally acuminate, and outer arms wider in the middle, narrowing at the ends, posteriorly shaped like a bird’s head; posterior pair, small, curved outwards, distally acuminate (Fig. 32). Paraprocts broad, almost triangular, sensory fields with 20–31 trichobothria on basal rosettes, setae as illustrated (Fig. 33). Epiproct trapeziform, wide anteriorly, with sides converging towards a straight posterior border, three mesal setae near anterior border, other setae as illustrated (Fig. 33).

#### Measurements

(in microns). FW: 3804, HW: 2569, F: 947, T: 1511, t1: 643, t2: 65, t3: 125; f1: 478, f2: 308, IO: 453, D: 328, d: 219, PO: 0.67.

### Key to the males of *Triplocania* of Subgroup MSB2, modified from Silva–Neto et al. (2014)

**Table:** 

1	Hypandrium of four sclerites	**6**
–	Hypandrium of no more than three sclerites	**2**
2	Hypandrium of a single sclerite	**3**
–	Hypandrium of three sclerites	**4**
3	Hypandrium with a long acuminate projection posteriorly, one projection on each antero-lateral extreme, deeply concave in outer margin, forming two acuminate projections, posterior endophallic sclerites with four acuminate projections each, one mesal and three distal	***Triplocania newi* Silva–Neto, Rafael & García Aldrete**
–	Hypandrium with a short acuminate projection posteriorly, one projection on each antero-lateral extreme, deeply cleft in the middle, posterior endophallic sclerites with three acuminate projections each	***Triplocania calcarata* New**
4	Central sclerite of hypandrium with five acuminate projections, side struts fused to external parameres	***Triplocania furcata* New**
–	Central sclerite of hypandrium with two projections, side struts not fused to external parameres	**5**
5	Central sclerite of hypandrium flanked by two large, almost triangular sclerites; posterior projections leaving a wide concavity between them; distal ends of posterior endophallic sclerites acuminate, paraprocts triangular	***Triplocania mariateresae* Silva–Neto, Rafael & García Aldrete**
–	Central sclerite of hypandrium flanked by two small, elongate sclerites; posterior projections leaving a narrow concavity between them; distal ends of posterior endophallic sclerites blunt, paraprocts semi-elliptic	***Triplocania plaumanni* Silva–Neto, Rafael & García Aldrete**
6	Posterior sclerite of hypandrium thicker in the middle, with posterior projection longer than the anterior-posterior length of the anterior sclerite; sickle-shaped lateral projections of the anterior sclerite barely reaching the inner margins of the lateral sclerite	***Triplocania lamasoides* sp. n.**
–	Posterior sclerite of hypandrium slender in the middle, with posterior projection not longer than the anterior-posterior length of the anterior sclerite; sickle-shaped lateral projections distal of the anterior sclerite surpass the inner margins of the lateral sclerites	***Triplocania lamasi* Silva–Neto, Rafael & García Aldrete**

## Discussion

*Triplocania bravoi* and *Triplocania erwini*, are the first species of *Triplocania* described from Ecuador. The hypandrium with side sclerites fused proximally to the central piece in *Triplocania bravoi* is an exceptional character within *Triplocania*; this character also appears in several species of *Loneura* Navás (*Loneura amazonica* (New), *Loneura erwini* (New & Thornton), *Loneura gorgonaensis* García Aldrete, González & Sarria, *Loneura insularis* García Aldrete, González & Sarria, and *Loneura monticola* García Aldrete, González & Sarria). Another exceptional character of *Triplocania bravoi* is the presence of a phallobase. Recently one of us (AMSN) examining specimens of *Triplocania magnifica* Roesler, noted the presence of a phallobase not described in the original paper by Roesler. The pattern of pigmentation and wing venation in *Triplocania bravoi* is similar to *Triplocania magnifica*, with small differences, but the hypandrium and phallosome structures of the two species are quite different.

*Triplocania trifida* and *Triplocania lamasoides* increase the diversity of *Triplocania* in Brazil to 16 species, this country being the most species rich so far for described species of *Triplocania*.

The transverse bridge in the phallosome of *Triplocania trifida* is a character that distinguishes it from other species of *Triplocania*; this character also appears in some species of *Loneura* (*Loneura jinotegaensis* García Aldrete, *Loneura mirandaensis* García Aldrete, *Loneura tuluaensis* García Aldrete, Mendivil & González, and *Loneura andina* García Aldrete, Mendivil & González. The structure the phallosome of *Triplocania trifida* is also very similar, except for the bridge, to the phallosome of *Loneura gorgonaensis*. The pattern of pigmentation and wing venation in *Triplocania trifida* is similar to *Triplocania erwini* with small differences, but the hypandrium and phallosome structures of the two species are different.

The remarkable similarities of phallosome and hypandrium in species of *Triplocania* and *Loneura* may indicate that the two genera are closer than previously thought.

*Triplocania lamasoides* and *Triplocania lamasi* constitute a pair of sister species within *Triplocania*. The morphological structure that separate them, are the lenght of the posterior projections of the anterior and posterior sclerite of the hypandrium. With more knowledge on the diversity of *Triplocania*, perhaps new cases of species complexes will be found, possibly confirming that the hypandrium is the most variable structure in *Triplocania*. The pair of species *Triplocania lamasoides*–*Triplocania lamasi* alerts also on the difficulty of association between males and females in *Triplocania*. The female of *Triplocania lamasoides* is the first female described for the subgroup MSB2, it was associated with the male because they were collected in the same place and date, but the wings and patterns of body pigmentation are also identical to *Triplocania lamasi*.

## Supplementary Material

XML Treatment for Triplocania bravoi

XML Treatment for Triplocania erwini

XML Treatment for Triplocania lamasoides

XML Treatment for Triplocania trifida
